# Supplementary material for: Gain and loss of acquired doxycycline resistance in Lactiplantibacillus plantarum: an adaptive laboratory evolution study
Source: Microbiol Spectr. 2026 Sep 18;14(11):e00892-26. doi: 10.1128/spectrum.00892-26 (PMC13729096; doi:10.1128/spectrum.00892-26)
Supplement: Supplementary file 1 [file reviewer-comments.pdf]

ASM | Microbiology Spectrum

Gain and Loss of Acquired Doxycycline Resistance in *Lactiplantibacillus plantarum*: An Adaptive Laboratory Evolution Study

Bryce Pierce, Olivia Harrison, Brooks Floyd, Agnes Moriarty, Kwadwo Antwi-Fordjour, Robert Hataway, and Brad Bennett

Corresponding Author(s): Brad Bennett, Samford University

Review Timeline:

Submission Date:	March 19, 2026
Editorial Decision:	June 11, 2026
Revision Received:	August 7, 2026
Accepted:	August 22, 2026

Editor: Cheryl Andam

Reviewer(s): Disclosure of reviewer identity is with reference to reviewer comments included in decision letter(s). The following individuals involved in review of your submission have agreed to reveal their identity: J. Jeffrey Morris (Reviewer #1)

Transaction Report:

(Note: With the exception of the correction of typographical or spelling errors that could be a source of ambiguity, letters and reports are not edited. The original formatting of letters and referee reports may not be reflected in this compilation.)

DOI: https://doi.org/10.1128/spectrum.00892-26

Re: Spectrum00892-26 (**Gain and Loss of Acquired Doxycycline Resistance in *Lactiplantibacillus plantarum*: An Adaptive Laboratory Evolution Study**)

Dear Dr. Brad C Bennett:

Thank you for the privilege of reviewing your work. Below you will find my comments, instructions from the Spectrum editorial office, and the reviewer comments.

Please return the manuscript within 60 days; if you cannot complete the modification within this time period, please contact me. If you do not wish to modify the manuscript and prefer to submit it to another journal, notify me immediately so that the manuscript may be formally withdrawn from consideration by Spectrum.

Revision Guidelines
To submit your modified manuscript, log into the submission site at https://spectrum.msubmit.net/cgi-bin/main.plex. Go to Author Tasks and click the appropriate manuscript title to begin. The information you entered when you first submitted the paper will be displayed; update this as necessary. Note the following requirements:

• Upload point-by-point responses to the issues raised by the reviewers in a file named "Response to Reviewers," NOT in your cover letter.
• Upload a compare copy of the manuscript (without figures) as a "Marked-Up Manuscript" file.
• Upload a clean .DOC/.DOCX version of the revised manuscript and remove the previous version.
• Each figure must be uploaded as a separate, editable, high-resolution file (TIFF or EPS preferred), and any multipanel figures must be assembled into one file.
• Any supplemental material intended for posting by ASM should be uploaded with their legends separate from the main manuscript. You can combine all supplemental material into one file (preferred) or split it into a maximum of 10 files with all associated legends included.

For complete guidelines on revision requirements, see our Submission and Review Process webpage. Submission of a paper that does not conform to guidelines may delay acceptance of your manuscript.

Data availability: ASM policy requires that data be available to the public upon online posting of the article, so please verify all links to sequence records, if present, and make sure that each number retrieves the full record of the data. If a new accession number is not linked or a link is broken, provide Spectrum production staff with the correct URL for the record. If the accession numbers for new data are not publicly accessible before the expected online posting of the article, publication may be delayed; please contact production staff (Spectrum@asmusa.org) immediately with the expected release date.

Publication Fees: For information on publication fees and which article types are subject to charges, visit our website. If your manuscript is accepted for publication and any fees apply, you will be contacted separately about payment during the production process; please follow the instructions in that e-mail. Arrangements for payment must be made before your article is published.

ASM Membership: Corresponding authors may join or renew ASM membership to obtain discounts on publication fees. Need to upgrade your membership level? Please contact Customer Service at Service@asmusa.org.

The ASM Journals program strives for constant improvement in our submission and publication process. Please tell us how we can improve your experience by taking this quick Author Survey.

Thank you for submitting your paper to Spectrum.

Sincerely,
Cheryl Andam
Editor
Microbiology Spectrum

Reviewer #1 (Comments for the Author):

This was an interesting study exploring the ability of fairly low sublethal antibiotic exposure to select for antibiotic resistance in a probiotic lactic acid bacterium. The study is well designed and documented and some of the results -- especially the analysis of candidate beneficial mutations and their structural effects -- are very thorough and compelling. That being said I felt there were a couple of serious missed opportunities that the authors can shore up with what I hope are some fairly straightforward additional

experiments using their cryopreserved cultures. There are also some issues (common with these kinds of experiments) in the evolutionary interpretation of some of the genetic results that need to be looked at again.

1. First, I would like to encourage the authors to include line numbers with their next version of this article. There were some small stylistic and grammatical fixes I noticed but without line numbers it would be too difficult to show where they are.

2. The authors need to be more clear about how frequently evolving cultures were cryopreserved, and how they were "resurrected" for downstream analysis and sequencing. Specifically, it needs to clarified whether they were ever bottlenecked by isolating individual colonies, or if resurrections and DNA extractions were performed on bulk cultures without a bottleneck.

3. The above point is particularly important for understanding the sequencing data. If DNA was extracted from clones obtained post-bottleneck, then we are looking at what ought to be clonal, haploid genomes. If on the other hand DNA was extracted from bulk cultures --as I suspect was the case -- then we are actually looking at metagenomes, as evolving cultures are mixtures of various mutants climbing up from single cells to possible ultimate fixation.

4. If I am correct that metagenomes were sequenced, was any thought given to variability or clonal interference within lineages? In other words, was there a minimum frequency cutoff a candidate variant had to cross before it was called as a variant as opposed to a sequencing error? If that value was higher than about 10%, there is likely a LOT of relevant genetic variation in the sequences that was overlooked. Basically, any mutation that has risen to detectability in an evolving metagenome is likely to be an adaptive mutation (otherwise, it would have to have reached a population size of millions by pure chance/drift).

5. This brings up a few issues with Table 3. First, which replicate lineage each mutation was observed in should be recorded. Ideally, these results would be presented as something like a phylogenetic tree or a Muller plot, showing WHEN certain mutations appear and whether or not they persist through time. If the authors do in fact have metagenome data, they could show a very nice Muller plot for each of the six lineages.

6. Also in Table 3, the terms "homozygous" and "heterozygous" are inappropriate; these are haploid bacteria! If there is a mixture of WT and mutant alleles, this likely represents an actual mixture of different kinds of cells - and hopefully it means these are sequenced metagenomes, because of they are sequenced clones it becomes very difficult to understand how to interpret such a result.

7. "Surprisingly, the Gen1200 sequences revealed 24 unique SNVs that did not overlap variants identified in the ALE." - why is this surprising? They adapted to a particular culture regime for 1200 generations, based on the LTPE that's long enough for several mutations to fix even without targeted selection.

8. The authors indicate that the loss of resistance after the removal of doxy pressure happened very quickly. Indeed, this is VERY hard to understand if adaptive mutations had fixed in the population, as the loss of resistance would require the chance appearance of a reversion mutation, its emergence out of the "drift zone" during its initial rarity, and then its rather arduous competitive climb to fixation as it displaces the resistance background strain.

9. An alternative, much more likely explanation for the rapid loss of resistance, is that the resistant variants had not yet fully supplanted their ancestors (see comments about metagenomes above). Clonal interference (see Gerrish and Lenski 1998, "The fate of competing beneficial mutations in an asexual population") can dramatically slow down the rate of fixation because of competition between multiple adaptive mutants with similar relative fitnesses. The rate of fixation would also be slower because of the relatively weak selection being applied compared to comparable evolution experiments (i.e., Table 1). If the candidate beneficial mutations were not fixed by 1000 generations, and there is a substantial fitness cost to resistance, then it's not that surprising that the susceptible mutants would rapidly displace them.

10. Which brings me to the issue of relatively straightforward culture experiments that I think would dramatically improve the manuscript. The authors could easily measure the cost of resistance, which is a very important undecided question in the study of antibiotic resistance, by competing evolved clones vs. the preserved ancestor (e.g., as in the Lenski experiments) either in the presence of, or the absence of, doxy, using the doxy resistance of the evolved clone as a selectable marker. The cost of resistance would just be relative fitness of the evolved strain WITH doxy minus the relative fitness of the evolved strain WITHOUT doxy. This could even be done with the specific clones that the authors have already confirmed the rpsJ genotype for, pinning costs pretty convincingly to specific mutations.

11. Also - IC50 experiments with the evolved clones containing confirmed rpsJ mutations would categorically link various resistance levels to various mutations, eliminating a lot of the guesswork in the discussion. (e.g. extensive discussion of POSSIBLE influences of structural changes on resistance profiles). Given the splendid analysis of the structural impact of the rpsJ mutations, this would be a really powerful addition that I hope is worth the effort.

12. A strange observation that really also needs some analysis, is the parallel change in resistance observed in the control

cultures (Fig. 2). Particularly weird is how it decreases when doxy is removed from the treatment lines -- but why would that have any impact on cultures that had never been treated with doxy at all? Is there any possibility of doxy carryover between the treatment groups somehow? Methodological changes in how the resistance assays were performed?

13. The discussion section could probably stand to have some sub-headings.

14. Is the data in Table 2 the same data shown in Fig 2? If so there's no need for a table as well; just put the error bars in the figure.

15. Are the plots on the right side of Figure 1 real data? If so, there should be error bars, or at least multiple replicate points per concentration tested - the model was fit with the raw data, not the averaged values, right?

16. Figure 2 also needs error bars. Also - put in an arrow indicating where doxy treatment was removed.

Reviewer #2 (Comments for the Author):

The manuscript describes a fairly simple study concerning the Lactiplantibacillus plantarum adaptive response to long term serial subculturing in the presence of a sub-lethal concentration of doxycycline.
The writing style and presentation are unusual. The manuscript is very long and contains considerable speculation. It reads more like a chapter in a traditional format PhD thesis than a journal article. The is considerable scope for making it more concise and focussed.
I have difficulty understanding the overarching rationale. It is true that ingestion of antibiotics by humans and animals selects resistance, and this is a bad thing. This is an unarguable truth that has been recognized for many decades. However, the actual experiment described here doesn't seem to ask a critical question related to this. A general model is that populations of opportunistic pathogens will be selected by antibiotic use to encompass resistant strains. The concept can be extended to commensals, with the significance being that commensals can harbour resistance specifying mobile elements with the potential to be transmitted to pathogens. This study doesn't seem to relate to these scenarios because L. plantarum is not a pathogen, and there was no consideration as to whether any resistance determinants identified would be transmissible (i.e. there appeared to be an assumption that the strain used was devoid of resistance markers, and there was no way in this experimental system that the bacteria could acquire transmissible resistance genes from anything else.) To put this another way, this experiment was only ever going to reveal de-novo chromosomal mutations in a non-pathogenic species. I guess chromosomal mutations on nominally non-mobile genes can potentially be mobilised into pathogens, but this is about the least likely scenario to lead to expansion of clinically significant resistance. In summary I would like to see a more specific and convincing justification for this study. (I am aware that something similar to this point is made in the Discussion, where it seems something of an afterthought.)
An obvious next step is to test the effects of the rpsJ mutations by creating genetically modified strains - and I think that really should be done for this article, given the focus on that gene. I am not sure whether this L. plantarum strain is amenable to allele replacement, but if it is not then that is a very significant strategic error by this team, given that the only way firm conclusions can be drawn from this type of experiment is to move on to testing of the effects of mutations using isogenic pairs.
Much of the study is arbitrary. Why was this strain of L. plantarum selected? I have already mentioned how essential it is that the strain should be amenable to genetic modification. Why not use an actual probiotic strain, given the prominence of the field of probiotics in the justifications for the study? Why was the sublethal concentration used 10X lower that the MIC? Why not 5X or 2X? That easily could have provided different (and more interesting) results. Also, why only a 10X dilution for every subculture. This only allows <4 generations before reaching stationary phase again. (Also, I think this figure makes the estimation of 1000 generations incorrect.)
One aspect of the methods really surprised me. Why were the culture tubes incubated with shaking. As a lactic acid bacterium, this species has a fermentative metabolism. It is true that L. plantarum can make cytochromes and respire if given haem and menaquinone, but the researchers did not do this. Incubating with shaking in unmodified MRS medium simply makes no sense- it is putting the cultures into oxidative stress for no reason, and making the experiment less relevant to what happens in the gut.
Table 3 should include whether the SNVs and synonymous or non-synonymous, the locations within coding sequences, and (if applicable) the amino-acid substitution. Doing this properly would make Table 4 redundant.
The results shown in Fig 2 are interesting, with respect to high IC50 reached and the very fast speed that the resistance disappears when selection is removed. This is hard to explain, and could have been looked at more closely. In other words, how is the increase in IC50 explained when such a low concentration of doxycycline is used? Taking the results at face value and stating them is simple terms - growing this strain in 5 µg/mL doxycycline selects a more than doubling of IC50 up to 70 µg/mL. Taking that 5 µg/mL doxycycline away again leads to an immediate IC50 drop to about 15. I guess this is possible but it just seems really odd.
Questions could include:
• Is copy number variation a factor? The data already obtained could be reanalysed to answer that. Copy number expansions could collapse at high frequency.
• What are the growth rates of mutants and WT, with and without the doxycycline? I know the IC50 experiments looks at something similar, but actually determining growth rates would have been illuminating.
Abstract
Line 12 should be '....non-synonymous mutation...". Same mistake in fourth last line of Introduction.

Response to Reviewers

Microbiology Spectrum Manuscript Number: Spectrum00892-26

Pierce et al. "Gain and Loss of Acquired Doxycycline Resistance in *Lactiplantibacillus plantarum*: An Adaptive Laboratory Evolution Study"

We thank the reviewers for their thoughtful and detailed critique of the manuscript. Their inquiries, suggestions, and advice have definitively improved the manuscript. We have used "Track changes" to indicate where our edits have been added, and we have added line numbers to make it easier to find specific edits.

Our responses are in italics.

Reviewer #1 (Comments for the Author):

This was an interesting study exploring the ability of fairly low sublethal antibiotic exposure to select for antibiotic resistance in a probiotic lactic acid bacterium. The study is well designed and documented and some of the results -- especially the analysis of candidate beneficial mutations and their structural effects -- are very thorough and compelling. That being said I felt there were a couple of serious missed opportunities that the authors can shore up with what I hope are some fairly straightforward additional experiments using their cryopreserved cultures. There are also some issues (common with these kinds of experiments) in the evolutionary interpretation of some of the genetic results that need to be looked at again.

1. First, I would like to encourage the authors to include line numbers with their next version of this article. There were some small stylistic and grammatical fixes I noticed but without line numbers it would be too difficult to show where they are.

We have added line numbers to help with identifying where any edits need to be made.

2. The authors need to be more clear about how frequently evolving cultures were cryopreserved, and how they were "resurrected" for downstream analysis and sequencing. Specifically, it needs to clarified whether they were ever bottlenecked by isolating individual colonies, or if resurrections and DNA extractions were performed on bulk cultures without a bottleneck.

3. The above point is particularly important for understanding the sequencing data. If DNA was extracted from clones obtained post-bottleneck, then we are looking at what ought

to be clonal, haploid genomes. If on the other hand DNA was extracted from bulk cultures --as I suspect was the case -- then we are actually looking at metagenomes, as evolving cultures are mixtures of various mutants climbing up from single cells to possible ultimate fixation.

This is a combined response for critiques #2-3. We especially thank the reviewer for these questions and points. We vacillated on whether we should attempt to purify a clonal isolate prior to DNA extraction and sequencing or whether we should just extract genomic DNA from bulk cultures. In our early discussions, we actually considered doing both and comparing sequencing results between a bottlenecked isolate and bulk culture. We ended up just extracting and getting sequencing data from bulk cultures. We agree that the sequencing data is thus actually metagenomic in nature, as many different populations are present in the bulk cultures. We have added several mentions of this in the text, including in Materials and Methods (lines 231-241, 303-305, 358-360), Results (lines 558-560), and Discussion (lines 813-816).

4. If I am correct that metagenomes were sequenced, was any thought given to variability or clonal interference within lineages? In other words, was there a minimum frequency cutoff a candidate variant had to cross before it was called as a variant as opposed to a sequencing error? If that value was higher than about 10%, there is likely a LOT of relevant genetic variation in the sequences that was overlooked. Basically, any mutation that has risen to detectability in an evolving metagenome is likely to be an adaptive mutation (otherwise, it would have to have reached a population size of millions by pure chance/drift).

Indeed, this is a very important threshold to consider, as it could inadvertently cause one to ignore/not know of potential variability. We used GATK to produce variant call format (VCF) files that list SNVs, Indels, CNVs, and structural variations when comparing a reference genome to a sequenced experimental genome. GATK essentially won't list an SNV if the reference (wild type) nucleotide is called >95% at a particular position. We empirically found from our data sets that this threshold is closer to 93%, meaning that the minimum frequency cutoff for a candidate variant to be listed is 7%. As this is below a 10% threshold, we think this cutoff has allowed us to identify any important SNVs in the WGS data sets. We included text to describe this in the Materials and Methods, lines 425-427.

5. This brings up a few issues with Table 3. First, which replicate lineage each mutation was observed in should be recorded. Ideally, these results would be presented as

something like a phylogenetic tree or a Muller plot, showing WHEN certain mutations appear and whether or not they persist through time. If the authors do in fact have metagenome data, they could show a very nice Muller plot for each of the six lineages.

Thank you to the reviewer for this suggestion. We have generated two Muller plots, one for the rpsJ SNVs identified by WGS alone and one for rpsJ SNVs observed by WGS and Sanger sequencing. We now include these plots as Supplemental Figure 1. Text has been added to the Materials and Methods section (lines 469-471) and to the Results section describing the plots, lines 610-612.

6. Also in Table 3, the terms "homozygous" and "heterozygous" are inappropriate; these are haploid bacteria! If there is a mixture of WT and mutant alleles, this likely represents an actual mixture of different kinds of cells - and hopefully it means these are sequenced metagenomes, because of they are sequenced clones it becomes very difficult to understand how to interpret such a result.

We agree that the use of these terms is problematic. And, yes, a "heterozygous" mixture in this context is actually a mixture of WT (reference) and mutant (alternate or variant alleles). As noted above, we used GATK to produce variant call format (VCF) files that list SNVs, Indels, CNVs, and structural variations when comparing a reference genome to a sequenced experimental genome. These files list the genotype ("GT") at a given nucleotide position as "homozygous reference" (0/0, where the reference allele or nucleotide is called), "homozygous alternate" (0/1, where the alternate allele or nucleotide is called), or "heterozygous" (1/1, where both the reference allele and the alternate allele are called). However, for bacteria, we agree that this is confusing. We have removed the terms homozygous and heterozygous from the formerly Table 3 (now Table 2) caption.

7. "Surprisingly, the Gen1200 sequences revealed 24 unique SNVs that did not overlap variants identified in the ALE." - why is this surprising? They adapted to a particular culture regime for 1200 generations, based on the LTPE that's long enough for several mutations to fix even without targeted selection.

Agreed. We have omitted "Surprisingly,". See line 573.

8. The authors indicate that the loss of resistance after the removal of doxy pressure happened very quickly. Indeed, this is VERY hard to understand if adaptive mutations had fixed in the population, as the loss of resistance would require the chance appearance of

a reversion mutation, its emergence out of the "drift zone" during its initial rarity, and then its rather arduous competitive climb to fixation as it displaces the resistance background strain.

9. An alternative, much more likely explanation for the rapid loss of resistance, is that the resistant variants had not yet fully supplanted their ancestors (see comments about metagenomes above). Clonal interference (see Gerrish and Lenski 1998, "The fate of competing beneficial mutations in an asexual population") can dramatically slow down the rate of fixation because of competition between multiple adaptive mutants with similar relative fitnesses. The rate of fixation would also be slower because of the relatively weak selection being applied compared to comparable evolution experiments (i.e., Table 1). If the candidate beneficial mutations were not fixed by 1000 generations, and there is a substantial fitness cost to resistance, then it's not that surprising that the susceptible mutants would rapidly displace them.

This response is for critiques #8-9. We agree that it is likely clonal interference and competition amongst mutants that allows the loss of DOX resistance to occur so quickly. The ability of a specific reversion mutation to emerge and displace at such as rapid rate is unlikely. We have added a section in the Discussion section to address this, lines 796-822. We even used some of the phrasing provided by the reviewer and have incorporated a few new references including the important Gerrish and Lenski book chapter the reviewer cited.

10. Which brings me to the issue of relatively straightforward culture experiments that I think would dramatically improve the manuscript. The authors could easily measure the cost of resistance, which is a very important undecided question in the study of antibiotic resistance, by competing evolved clones vs. the preserved ancestor (e.g., as in the Lenski experiments) either in the presence of, or the absence of, doxy, using the doxy resistance of the evolved clone as a selectable marker. The cost of resistance would just be relative fitness of the evolved strain WITH doxy minus the relative fitness of the evolved strain WITHOUT doxy. This could even be done with the specific clones that the authors have already confirmed the rpsJ genotype for, pinning costs pretty convincingly to specific mutations.

11. Also - IC50 experiments with the evolved clones containing confirmed rpsJ mutations would categorically link various resistance levels to various mutations, eliminating a lot of the guesswork in the discussion. (e.g. extensive discussion of POSSIBLE influences of structural changes on resistance profiles). Given the splendid analysis of the structural

impact of the rpsJ mutations, this would be a really powerful addition that I hope is worth the effort.

This response is for critiques #10-11. Thank you for this idea, and we agree that these are important experiments needed for a better interpretation of the ALE and sequencing data. We have now performed growth curve experiments comparing generation 0 (the three ALE starting lineages) and generation 1000 lineages across a range of DOX, from 0 to 6.5 ug/mL (~1/4 MIC). There is a clear trend for Gen0 cultures outpacing the Gen1000 cultures with no or very little DOX in the media and then an inversion of this where Gen1000 outperforms Gen0 cultures at higher DOX concentrations. Furthermore, fitness tests with the Gen0 cultures give an IC50 of ~13 ug/mL, much lower than Gen1000 and on par with values measured for Gen1050 onwards in the original ALE experiment. Please see lines 212-217 (Introduction), 473-496 (Materials and Methods), 622-638 (Results), and 796-822 (Discussion) for a description of the growth curve experiments and interpretation of this data.

12. A strange observation that really also needs some analysis, is the parallel change in resistance observed in the control cultures (Fig. 2). Particularly weird is how it decreases when doxy is removed from the treatment lines -- but why would that have any impact on cultures that had never been treated with doxy at all? Is there any possibility of doxy carryover between the treatment groups somehow? Methodological changes in how the resistance assays were performed?

We agree with the reviewer that this is a strange phenomenon. We did notice this at the time of the experiment when performing the fitness tests and were concerned that some cross-contamination had occurred. However, we cannot confirm or deny this. The fitness tests were performed identically across the experiment. We thought maybe an rpsJ variant arose in a control culture, however, we didn't identify any of the typical DOX resistance SNVs (such as tet or rpsJ variants) in WGS data in control cultures across the ALE experiment. There are SNVs unique to generation 1200 controls, so there is a possibility that a variant arose in controls late in the experiment that positively contributed to growth (overall fitness) but adversely affected its fitness specifically to DOX and thus caused its IC_{50} to decrease between generation 1000 and the end of the experiment. A more thorough analysis of control culture changes as well as SNVs in generation 1200 WGS data for both control and DOX-exposed cultures is a future direction of the project.

13. The discussion section could probably stand to have some sub-headings.

We have added section sub-headings to the Discussion.

14. Is the data in Table 2 the same data shown in Fig 2? If so there's no need for a table as well; just put the error bars in the figure.

Yes, it is, however, the RMSEs listed in Table 2 are quite small and would be very difficult to see as error bars on the plot in Figure 2. Thus, we think an adequate compromise is to embed Table 2 as an inset within Figure 2. This way, the IC50 values and their errors are listed and can be directly compared to the plot. (Doing this will mean that Tables 3-5 will now be re-numbered to 2-4.)

15. Are the plots on the right side of Figure 1 real data? If so, there should be error bars, or at least multiple replicate points per concentration tested - the model was fit with the raw data, not the averaged values, right?

No, they (and colors used in the microplate wells) are just demo data so that the reader can visually see an example of the experiment layout and output data. We have included this as the last line of text in the figure caption.

16. Figure 2 also needs error bars. Also - put in an arrow indicating where doxy treatment was removed.

Regarding error bars, see our response to #14 above. Instead of an arrow, we just state in the caption when DOX was removed.

Reviewer #2 (Comments for the Author):

The manuscript describes a fairly simple study concerning the Lactiplantibacillus plantarum adaptive response to long term serial subculturing in the presence of a sub-lethal concentration of doxycycline.

The writing style and presentation are unusual. The manuscript is very long and contains considerable speculation. It reads more like a chapter in a traditional format PhD thesis than a journal article. The is considerable scope for making it more concise and focussed.

I have difficulty understanding the overarching rationale. It is true that ingestion of antibiotics by humans and animals selects resistance, and this is a bad thing. This is an unarguable truth that has been recognized for many decades. However, the actual experiment described here doesn't seem to ask a critical question related to this. A general model is that populations of opportunistic pathogens will be selected by antibiotic use to encompass resistant strains. The concept can be extended to commensals, with the significance being that commensals can harbour resistance specifying mobile elements with the potential to be transmitted to pathogens. This study doesn't seem to relate to these scenarios because L. plantarum is not a pathogen, and there was no consideration as to whether any resistance determinants identified would be transmissible (i.e. there appeared to be an assumption that the strain used was devoid of resistance markers, and there was no way in this experimental system that the bacteria could acquire transmissible resistance genes from anything else.) To put this another way, this experiment was only ever going to reveal de-novo chromosomal mutations in a non-pathogenic species. I guess chromosomal mutations on nominally non-mobile genes can potentially be mobilised into pathogens, but this is about the least likely scenario to lead to expansion of clinically significant resistance. In summary I would like to see a more specific and convincing justification for this study. (I am aware that something similar to this point is made in the Discussion, where it seems something of an afterthought.)

We appreciate the reviewer's suggestion for a more compelling justification. Though we are not clinicians, a broad interest of ours is how antibiotics may adversely affect the benefits of probiotics to its host. We are much less interested in: 1) how probiotics can in rare cases be pathogenic themselves and 2) how probiotics might become reservoirs for resistance genes and then share those with established pathogens. There's little (but not zero) concern for these possibilities amongst clinicians and medical microbiologists. Indeed, as you stated, our ALE experiment is not well-suited for lines of inquiry relating to L. plantarum becoming pathogenic or its ability to uptake ARGs from and/or share ARGs with its environment. To get back to the original broad point of the project, the interplay between probiotics and antibiotics, one application of this study would be in the use of <u>resistant</u> strains of probiotics as a supplement in antibiotic therapies, in order to mitigate the negative side effects of the antibiotic on gut health and stability. Admittedly, we have not seen much literature on this but are hopeful this could be the focus of controlled trials in the future. We have added text in a few places in the manuscript which hopefully better justifies, at least in an applied sense, the present study: see lines 113-114 and 217-221 in the Introduction and in the Discussion, interspersed within lines 851-863.

An obvious next step is to test the effects of the rpsJ mutations by creating genetically modified strains - and I think that really should be done for this article, given the focus

on that gene. I am not sure whether this L. plantarum strain is amenable to allele replacement, but if it is not then that is a very significant strategic error by this team, given that the only way firm conclusions can be drawn from this type of experiment is to move on to testing of the effects of mutations using isogenic pairs.

This strain can be used for chromosomal insertion of mutant rpsJ alleles (see lines 871 onward), and we have plans for this in the near future. We have modified the text at lines 883-886.

Much of the study is arbitrary. Why was this strain of L. plantarum selected? I have already mentioned how essential it is that the strain should be amenable to genetic modification. Why not use an actual probiotic strain, given the prominence of the field of probiotics in the justifications for the study?

We admit there are numerous notable and well-documented strains of L. plantarum, and many of them could have been selected for this study. The strain we ultimately selected is an ATCC type strain, 14917, also known as Lp 39, and is well studied. It is used as a quality control strain, its probiotic properties have been characterized, it has its whole genome sequenced, and it is referenced frequently in the probiotic literature. Lastly, this strain has been used in at least two ALE experiments where antibiotics were used as the selection pressure, so there is some precedent to investigating this strain and its resistance properties. We have added text (lines 227-229) in the Materials and Methods to better justify the use of this strain.

Why was the sublethal concentration used 10X lower that the MIC? Why not 5X or 2X? That easily could have provided different (and more interesting) results.

Yes, we agree that the choice of the sublethal concentration can definitely provide different results. Most ALE experiments do utilize concentrations closer to the MIC than we used. Indeed, a more stringent (higher) concentration of doxycycline, such as 2X lower than the MIC, could have caused a more rapid gain of resistance, with different or more numerous genetic variants underlying the mechanism of resistance. However, we are quite interested in probiotic response to weak selective pressure. Thus, we surveyed the literature for practical antibiotic concentration ranges used in ALE studies; again, most are 2-4X lower than the MIC, however, we found the range went from 2X to 20X lower than the MIC. Thus, we chose 10X below the MIC as within range of what is normal but on the less stringent and “weaker” end (lower concentration). We have added text on lines 292-294 (Materials and Methods) and 511-512 (Results) addressing this.

Also, why only a 10X dilution for every subculture. This only allows <4 generations before reaching stationary phase again. (Also, I think this figure makes the estimation of 1000 generations incorrect.)

For both the ALE and the fitness tests, we used a 5% inoculum, which is a 20X dilution. (In our measurements, this inoculum means the starting cultures are at an OD600 of ~0.05-0.1, which is a concentration of ~1.5 – 3 x 10^7 CFU/mL.) With a doubling time at ~4 hours, this provided time for 6-7 generations per day, ~45 generations per week. Our experimental generation number in the ALE is based off these observations, but we admit it is an estimate and could be 10-15% lower if the cultures consistently grew at the lower end of our doubling time measurement. Thus, generation 1000 could be closer to generation 900. We have included text to qualify this in the Materials and Methods section, on lines 261-262 and 299-301.

One aspect of the methods really surprised me. Why were the culture tubes incubated with shaking. As a lactic acid bacterium, this species has a fermentative metabolism. It is true that L. plantarum can make cytochromes and respire if given haem and menaquinone, but the researchers did not do this. Incubating with shaking in unmodified MRS medium simply makes no sense- it is putting the cultures into oxidative stress for no reason, and making the experiment less relevant to what happens in the gut.

This was an oversight and has been omitted.

Table 3 should include whether the SNVs and synonymous or non-synonymous, the locations within coding sequences, and (if applicable) the amino-acid substitution. Doing this properly would make Table 4 redundant.

We have now included a column in Table 3 that includes whether a mutation is silent or missense, the amino acid position, and the amino acid change (if any). In the Results, we have added lines 563-565 detailing the number of missense and silent mutations that would be the consequence of the unique SNVs identified in the WGS. We wish to keep table 4 as it focuses on rpsJ specifically and provides the location in the S10 protein structure where the mutation resides.

The results shown in Fig 2 are interesting, with respect to high IC50 reached and the very fast speed that the resistance disappears when selection is removed. This is hard to explain, and could have been looked at more closely. In other words, how is the increase in IC50 explained when such a low concentration of doxycycline is used? Taking the results at face value and stating them is simple terms - growing this strain in 5 µg/mL doxycycline selects a more than doubling of IC50 up to 70 µg/mL. Taking that 5 µg/mL

doxycycline away again leads to an immediate IC50 drop to about 15. I guess this is possible but it just seems really odd.

We were not so surprised with the gradual increase in the IC_{50}; a driving point of the manuscript is that weak pressure is able to force resistance, though modest. However, we were surprised the IC_{50} collapsed as quickly as it did after removal of the DOX. We have added some explanation, mostly from an rpsJ variant perspective, in the Discussion (lines 796-822).

Questions could include:

• Is copy number variation a factor? The data already obtained could be reanalysed to answer that. Copy number expansions could collapse at high frequency.

Copy number and structural variation are two components provided in the WGS data that we plan to analyze next in this project.

• What are the growth rates of mutants and WT, with and without the doxycycline? I know the IC50 experiments looks at something similar, but actually determining growth rates would have been illuminating.

Thank you for this idea, and we agree that these are important experiments needed for a better interpretation of the ALE and sequencing data. We have now performed growth curve experiments comparing generation 0 (the three ALE starting lineages) and generation 1000 lineages across a range of DOX, from 0 to 6.5 ug/mL (~1/4 MIC). There is a clear trend for Gen0 cultures outpacing the Gen1000 cultures with no or very little DOX in the media and then an inversion of this where Gen1000 outperforms Gen0 cultures at higher DOX concentrations. Please see lines 212-217 (Introduction), 473-496 (Materials and Methods), 622-638 (Results), and 796-822 (Discussion) for a description of the growth curve experiments and interpretation of this data.

Abstract

Line 12 should be '....non-synonymous mutation...". Same mistake in fourth last line of Introduction.

Thank you. These were typos, and we actually fixed both after submitting the original manuscript but before receiving the reviews. Corrections are found on line 55 (Abstract) and line 209 (Introduction).

Re: Spectrum00892-26R1 (**Gain and Loss of Acquired Doxycycline Resistance in *Lactiplantibacillus plantarum*: An Adaptive Laboratory Evolution Study**)

Dear Dr. Brad C Bennett:

Your manuscript has been accepted, and I am forwarding it to the ASM production staff for publication. Your paper will first be checked to make sure all elements meet the technical requirements. ASM staff will contact you if anything needs to be revised before copyediting and production can begin. Otherwise, you will be notified when your proofs are ready to be viewed.

Data Availability: ASM policy requires that data be available to the public upon online posting of the article, so please verify all links to sequence records, if present, and make sure that each number retrieves the full record of the data. If a new accession number is not linked or a link is broken, provide production staff with the correct URL for the record. If the accession numbers for new data are not publicly accessible before the expected online posting of the article, publication may be delayed; please contact ASM production staff immediately with the expected release date.

Publication Fees: For information on publication fees and which article types have charges, please visit our website. We have partnered with Copyright Clearance Center (CCC) to collect author charges. If fees apply to your paper, you will receive a message from no-reply@copyright.com with further instructions. For questions related to paying charges through RightsLink, please contact CCC at ASM_Support@copyright.com or toll free at +1-877-622-5543. CCC makes every attempt to respond to all emails within 24 hours.

ASM Membership: Corresponding authors may join or renew ASM membership to obtain discounts on publication fees. Need to upgrade your membership level? Please contact Customer Service at Service@asmusa.org.

Embargo Policy: A press release may be issued as soon as the manuscript is posted on the Spectrum Latest Articles webpage. The corresponding author will receive an email with the subject line "ASM Journals Author Services Notification" when the article is available online.

Cover Image Submissions: If you would like to submit a potential Cover Image, please email a file and a short legend to spectrum@asmusa.org. Please note that we can only consider images that (i) the authors created or own and (ii) have not been previously published. By submitting, you agree that the image can be used under the same terms as the published article. Image File requirements: TIF/EPS, 7.5 inches wide by 8.25 inches tall (at least 2,250 pixels wide by 2,475 pixels tall), minimum 300 dpi resolution (600 dpi preferred), RGB, and no figure elements, e.g., arrows or panel labels. The legend should be a short description of the image, 1-2 sentences recommended. Please use the ASM Journals Cover Image Sizing Tool (https://journals.asm.org/pb-assets/html/asm-cover-sizing-tool.html) to ensure that your proposed cover image meets our size requirements.

The ASM Journals program strives for constant improvement in our submission and publication process. Please tell us how we can improve your experience by taking this quick Author Survey.

Thank you for submitting your paper to Spectrum.

Sincerely,
Cheryl Andam
Editor
Microbiology Spectrum

Reviewer #1 (Comments for the Author):

The authors have done an admirable job fielding my previous comments. In particular, their inclusion of fitness assays confirming the cost of DOX resistance are very welcome. I have no further concerns with the manuscript.
