# Supplementary material for: Gain and loss of acquired doxycycline resistance in Lactiplantibacillus plantarum: an adaptive laboratory evolution study
Source: Microbiol Spectr. 2026 Sep 18;14(11):e00892-26. doi: 10.1128/spectrum.00892-26 (PMC13729096; doi:10.1128/spectrum.00892-26)
Supplement: Supplementary file 2 [file spectrum.00892-26-s0001.pdf]

Supplemental Information for:

Gain and Loss of Acquired Doxycycline Resistance in *Lactiplantibacillus plantarum*: An Adaptive Laboratory Evolution Study

Bryce Pierce, [a*] Olivia Love Harrison, [a*] Brooks Floyd, [a] Agnes Moriarty, [a] Kwadwo Antwi-Fordjour,[b] Robert A. Hataway,[a] Brad C. Bennett [a#]

[a]Department of Biological and Environmental Sciences, Samford University, Birmingham, AL, USA
[b]Department of Mathematics and Computer Science, Samford University, Birmingham, AL, USA

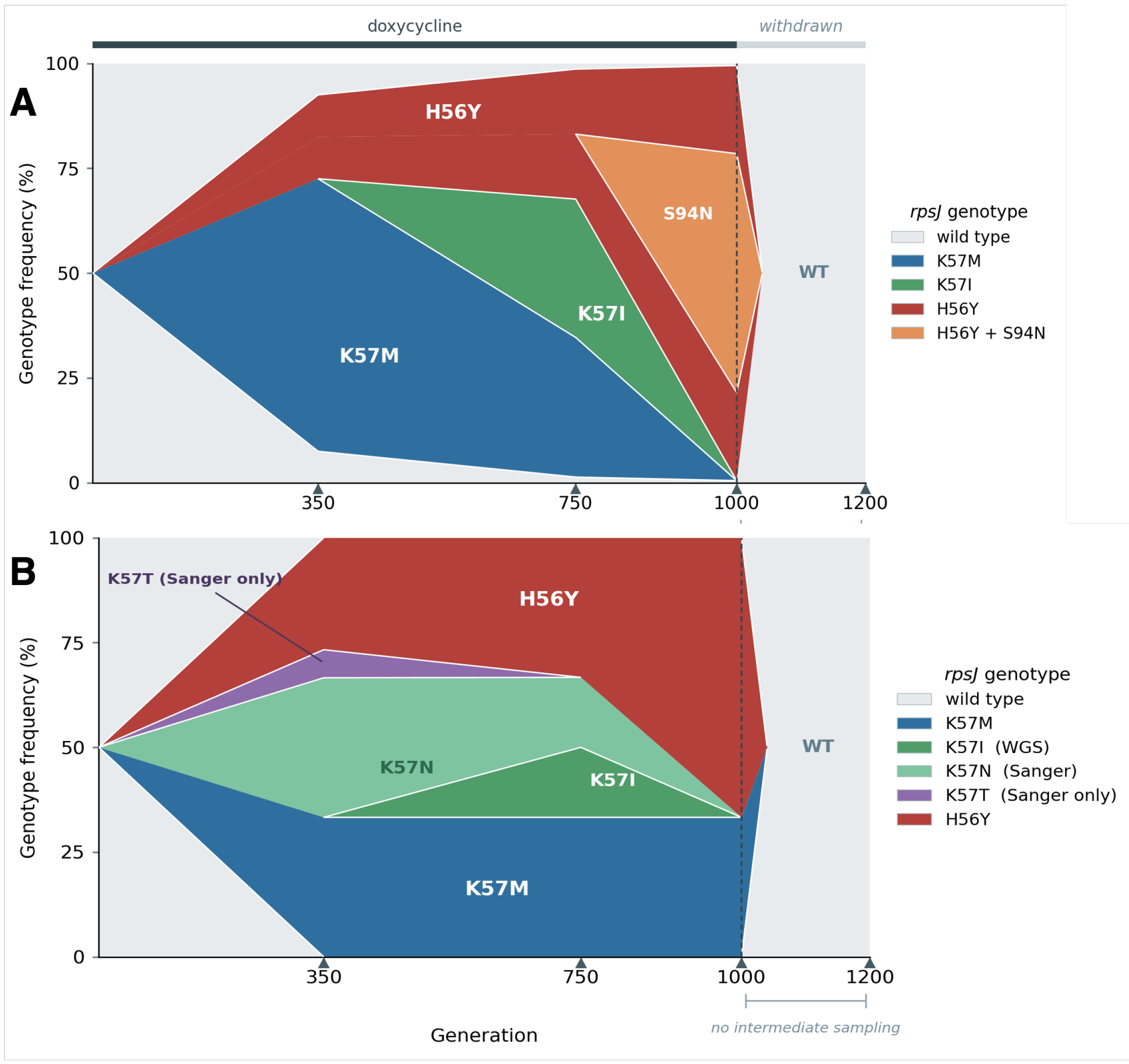

Supplemental Figure 1. Genotype dynamics of *rpsJ* variants revealed by whole genome and targeted Sanger sequencing. A) Muller plot showing the emergence and retreat of several *rpsJ* variants, based solely on whole genome sequencing (WGS) at the generations listed on the X-axis. The genotype frequency, shown as a percentage on the Y-axis, is based on the frequency of a given variant at a defined generation. Frequency was calculated by determining the fraction of reads at a particular position that carried the variant nucleotide call and was averaged across the number of DOX-exposed cultures that were sequenced at the defined generation. B) Muller plot as in (A), but this also includes variants identified by Sanger sequencing targeting the *rpsJ* gene. For the Sanger data, frequency is calculated based on proportion of colonies sequenced where a particular variant at a defined generation was identified. The S94N variant was only observed in the WGS, so it is omitted on this plot. At generation 1200, Sanger sequencing (but not WGS) revealed that one of the DOX-exposed cultures retained an H6Y variant, however, it is not represented on this plot. Plots were generated by an R-based program, ggmuller (https://CRAN.R-project.org/package=ggmuller). To determine and verify lineages with shared variants, such as H56Y and S94N in (A), we used lollipop (v0.9; https://github.com/cdeitrick/Lolipop).

Supplemental Table 1. Growth Metrics for *Lp14917* ALE Strains With and Without DOX

ALE generation/DOX (in μg/mL)	Average rate (hrs)	Max rate (hrs)	Average doubling time (hrs)	Fastest doubling time (hrs)
Gen0/No DOX	0.088	0.15	6.54	2.25
Gen0/0.4	0.052	0.092	8.32	2.57
Gen0/0.8	0.039	0.072	13.5	3.63
Gen0/1.6	0.027	0.070	14.8	3.20
Gen0/3.2	0.018	0.034	12.8	4.72
Gen0/6.5*	n.d.	n.d.	n.d.	n.d.
Gen1000/No DOX	0.077	0.205	6.24	3.75
Gen1000/0.4	0.054	0.100	8.70	3.18
Gen1000/0.8	0.048	0.081	8.68	3.10
Gen1000/1.6	0.053	0.121	6.88	3.07
Gen1000/3.2	0.031	0.059	7.63	4.49
Gen1000/6.5	0.016	0.031	15.0	13.1

*No Gen0 lineage grew in the presence of DOX at 6.5 μg/mL, thus, no growth metrics could be determined.
